# The case of Scott Ortiz: a clash between criminal justice and public health

**DOI:** 10.1186/1477-7517-3-21

**Published:** 2006-07-24

**Authors:** Homer D Venters, Asiya M Razvi, Maria S Tobia, Ernest Drucker

**Affiliations:** 1 Montefiore Medical Center, CHCC Clinic 305 E. 161^st ^St., Bronx N.Y. 10451, USA; 2 Bronx Defenders 860 Courtlandt Avenue, Bronx, N.Y. 10451, USA; 3 Montefiore Medical Center Department of Social and Family Medicine, 1300 Morris Park Avenue – Suite 100, Bronx NY, 10461, USA

## Abstract

The criminal justice system creates particular challenges for persons with HIV and Hepatitis C, many of whom have a history of injection drug use. The case of Scott Ortiz, taken from public trial and sentencing transcripts, reveals the manner in which incarceration may delay learning of important health problems such as Hepatitis C infection. In addition, the case of Mr. Ortiz suggests the bias in sentencing that a former injection drug user may face. Collaboration between the Montefiore Medical Center residency in Social Medicine and a Bronx legal services agency, Bronx Defenders, yielded the discovery that a decade after diagnosis with HIV and after long term incarceration, Mr. Ortiz was infected with Hepatitis C. Mr. Ortiz only became aware of his advanced Hepatitis C and liver damage during his trial. The second important aspect of this case centers on the justification for lengthy sentence for a burglary conviction. The presiding Judge in Mr. Ortiz's case acknowledged that because of his advanced illness, Mr. Ortiz posed no threat to society as a burglar (the crime for which he was convicted). But the Judge elected to use his discretion to sentence Mr. Ortiz to a term of 15 years to life (as opposed to a minimum of two to four years) based on the idea that the public health would be served by preventing Mr. Ortiz from returning to the life of a street addict, sharing dirty needles with others. Mr. Ortiz reports distant injection drug use, no evidence of current or recent drug use was presented during Mr. Ortiz's trial and he reports no injection drug use for over a decade. In this case, bias against a former injection drug user, masquerading as concern for public health, is used to justify a lengthier sentence. Mr. Ortiz's lack of awareness of his Hepatitis C infection despite long term incarceration, combined with the justification for his dramatically increased sentence, provide examples of how persons within the criminal justice system may face particular challenges to their health.

## Background

Involvement in the criminal justice system (CJS) creates particular difficulties for persons with HIV and Hepatitis C, many of whom have histories of injection drug use (IDU) and substantial criminal records [1]. The case of Mr. Scott Ortiz, as revealed in the public trial transcripts in the Bronx NY during 2005 and 2006, lays bare the conflicting agendas of public health and criminal justice and demonstrates how they may play out in ways that serve neither the individual nor public health and safety. This case also illustrates how the health care of such an individual may suffer when they are in hands of the CJS, in this instance in the guise of protecting the public not from crime, but from infectious disease. This case report flows from a new collaboration between Bronx Defenders (a public defender organization) and Montefiore Medical Center's residency program in Social Internal Medicine, in which medical, social work and legal professionals work together to understand the interface of law and health and advocate for better client outcomes. Both organizations are committed to advocacy in the South Bronx N.Y., an area blighted by poverty, disease, drug use and the decades-long war on drugs and are seeking to improve our understanding of the consequences of our populations recurrent involvement in the CJS, and its implications for individual and public health

## Case presentation

Mr. Ortiz, a 45-year old Bronx resident, was convicted of burglary in June 2005 [2]. A review of medical records during Mr. Ortiz' trial revealed that he was also Hepatitis C positive and that he had persistently low platelets for several years, irrespective of a fluctuating CD4+ level/HIV viral load. Although aware of his HIV infection since original diagnosis in the late 1990's, Mr. Ortiz was unaware of his hepatitis status until his trial when review of his records revealed a positive Hepatitis C test from a hospital visit after his most recent incarceration and shortly before his arrest on the burglary charge for which he was convicted. Mr. Ortiz reports regular medical care during his prior incarcerations, including diagnosis and treatment of his HIV but recalls no mention of liver disease [3]. This finding led Mr. Ortiz's defense team to seek further medical evaluation. Testimony by a consulting gastroenterologist was presented during the sentencing phase of the trial and identified Mr. Ortiz as having advanced Hepatitis C with platelets too low for biopsy [4]. Mr. Ortiz reports having used IV drugs 10–15 years prior to his recent trial which is consistent with the observation that approximately 70% of person's co-infected with HIV and Hepatitis C become infected via IVDU [5]. Given the slow progression of hepatitis C, even allowing for a hastened disease course in the setting of HIV infection, it is likely that Mr. Ortiz became infected at least ten years earlier. Over this period of time, Mr. Ortiz was known to have HIV and was being intermittently treated for HIV infection.

Aside from never learning of his Hepatitis C while incarcerated, another example of the burdens that the CJS may impose on individuals such as Mr. Ortiz is bias in sentencing. Because of his multiple criminal convictions, the Bronx District Attorney asked that the presiding Judge sentence Mr. Ortiz to a lengthy prison term as a 'persistent felony offender'. This rarely used statute allows a Judge to impose a sentence of 15 year to life, as opposed to whatever sentence would normally apply for their individual conviction [6]. In this case the burglary charge (even with the prior offenses) could have brought Mr. Ortiz as little as a 2 to 4 year sentence. Instead, the Judge held that Mr. Ortiz was a persistent felony offender and sentenced him to 15 years to life, noting that the "Extensive medical testimony and other evidence established that it is questionable whether, even if I sentence him merely as a second felony offender to the minimum sentence of 2 to 4 years, he would still be alive at the expiration of his sentence" (see Additional file 1). He went on to explain that "Given his weakened state, any assertion that the Defendant is a serious threat to the public as a burglar is really not credible. However, if released, he may well return to the life of a street addict, thereby endangering the public health through the exchange or sharing of dirty needles." This despite the fact that during this trial, no evidence was introduced about Mr. Ortiz currently or recently using intravenous drugs. Other trial testimony available to the Judge indicates that Mr. Ortiz had last reported intravenous drug use over a decade before his trial. Yet the Judge concluded that incarceration in a state prison would provide "a setting that will ensure that he takes his medications on schedule, and without resort to the use and sharing of dirty needles" [7].

## Conclusion

The case of Mr. Ortiz underscores two significant challenges that the criminal justice system creates for those with HIV and hepatitis C. First, with regard to Mr. Ortiz not knowing that he had hepatitis C, one can imagine multiple scenarios. Mr. Ortiz may not have been tested for hepatitis infection during his numerous incarcerations. Even if tested, and informed that he had Hepatitis C, this information may not have been explained to Mr. Ortiz in a manner that he understood. Recent reporting by the Correctional Association of New York (an independent, non-profit prison oversight organization founded in 1844) has shown that despite hepatitis C prevalence of 14% in incoming prison inmates, testing is often discouraged by prison staff and little education about hepatitis occurs among inmates. This same organization has documented cases in which inmates with hepatitis C were offered treatment only on the condition that the defendant stay in prison past their release date in order to complete a 12 month course of treatment [8]. The experience of Mr. Ortiz is consistent with these findings, for despite being intermittently treated for his HIV during incarceration, he appears either not to have been tested or not informed of his Hepatitis C. Because of the aggressive, irreversible nature of liver damage in HIV/Hepatitis C co-infection, Mr. Ortiz will unfortunately pay a dear price for this delay in diagnosis [9].

Second, by employing his concern for public health to impose a life sentence, the Judge in this case transforms unfounded assumptions about a former intravenous drug user into an ersatz public health intervention. The Judge reveals this bias by discounting any future threat by Mr. Ortiz as a burglar (due to his "*weakened state*") while raising the specter of a 'street addict' running amok in the community, "endangering the public health through the exchange or sharing of dirty needles". Not only does this Judge's assessment lack any supporting evidence of current or recent intravenous drug use by Mr. Ortiz, it trades on the bigoted and erroneous belief that removing people with substance abuse problems from our midst advances our collective public health and even solves the problem of their substance abuse. While the legal standards for application of the 'persistent felony offender' statute are outside the bounds of this review, the notion that using past drug use as a criterion for life incarceration out of concern for public health is both irrational and hypocritical, serving neither justice or public health.

This decision comes in the midst of efforts in New York to redress the damage done by the war on drugs, codified in the State's Rockefeller drug laws and their long mandatory sentences for drug users. Though widely reviled as cruel and ineffective, they have served as the templates for similar state and Federal laws that are at the root of the 10 fold growth of the US prison system over the last 30 years [10]. If Judges use past drug use as a reason to exercise their discretion towards longer sentences under the guise of 'safeguarding' the public health, then even advances made in legislative reform (lessening of mandatory sentences) and diversion to addiction treatment will surely be reversed, albeit one case at a time. Strong and able voices are needed to advocate for the health of those individuals who run afoul of the criminal justice system, such as Mr. Ortiz, both to safeguard their individual health, and to focus attention on the broader harms that the criminal justice system may visit upon public health of vulnerable populations. This process will be advanced by more of the type of collaborative arrangements between medical and legal advocates as we are developing in the Bronx.

## Competing interests

The author(s) declare that they have no competing interests.

## Authors' contributions

This manuscript was written by HV and ED. The actual work of defending Mr. Ortiz was performed by M.T. and A.R. served as social worker for his defense team. Both M.T. and A.R. provided copies of public trial and sentencing transcripts. In addition, A.R. and M.T. assisted in the editing and preparation of this manuscript.

## Supplementary Material

Additional file 1 A pdf file of the decision of the presiding Judge in the case of Mr. Ortiz is included. This file is named 'Scott Ortiz Decision.pdf'. Click here for file
