# Supplementary material for: The case of Scott Ortiz: a clash between criminal justice and public health
Source: Harm Reduct J. 2006 Jul 24;3:21. doi: 10.1186/1477-7517-3-21 (PMC1544323; doi:10.1186/1477-7517-3-21)
Supplement: Additional file 1 — A pdf file of the decision of the presiding Judge in the case of Mr. Ortiz is included. This file is named 'Scott Ortiz Decision.pdf'. [file 1477-7517-3-21-S1.pdf]

SUPREME COURT OF THE STATE OF NEW YORK
BRONX COUNTY : CRIMINAL DIVISION : PART T16

-- X

THE PEOPLE OF THE STATE OF NEW YORK, :

- against - :

SCOTT ORTIZ, :

Defendant. :

-- X

DECISION AFTER PERSISTENT FELONY OFFENDER HEARING

Ind. No. 3658-04

RICHARD LEE PRICE, J.:

Although the Defendant was found guilty by a jury of his peers of Burglary in the third degree, P.L. § 140.20, on June 29, 2005, this matter has remained on my calendar because the People have chosen to extensively present evidence that 1) the Defendant meets that statutory definition of a person eligible for sentencing as a persistent felony offender, ***see*** P.L. § 70.10[1], and 2) this Court should exercise its statutory discretion and so sentence him as if convicted of an "A" felony, as is permitted by subdivision 2 of P.L. § 70.10. Subdivision 2 of that statute provides

> 2. Authorized sentence. When the court has found, pursuant to the provisions of the criminal procedure law, that a person is a persistent felony offender, and when it is of the opinion that the history and character of the defendant and the nature and circumstances of his criminal conduct indicate that extended incarceration and life-time supervision will best serve the public interest, the court, in lieu of imposing the sentence of imprisonment authorized by section 70.00, 70.02, 70.04 or 70.06 for the crime of which such person presently stands convicted, may impose the sentence of imprisonment authorized by that section for a class A-I felony. In such event the reasons for the court's opinion shall be set forth in the record.

P.L. § 70.10[2]. The Court of Appeals has explained that the determination as to whether to sentence a defendant as a persistent felony offender is a purely discretionary decision. People v. Rivera, 5 N.Y.3d 61, 70 (2005) ("Once the defendant is adjudicated a persistent felony offender, the requirement that the sentencing justice reach an opinion as to the defendant's history and character is merely another way of saying that the court should exercise its discretion").

DISCUSSION

The hearing was conducted over lo!, these many months, with no less than 20 appearances. I conclude without any hesitation that the People have established, beyond a reasonable doubt, that the Defendant is eligible to be sentenced as a persistent felony offender because it is without peradventure that he stands convicted of a felony after previously being convicted of two other felonies, as provided in subdivisions (b) and (c) of P.L. § 70.10[1].

After full consideration of all of the evidence presented by both the People and Defendant's able counsel, and the excellent arguments presented by both counsel, I find that the People have met their burden of establishing "that the history and character of the defendant and the nature and circumstances of his criminal conduct indicate that extended incarceration and life-time supervision will best serve the public interest", as provided in P.L. § 70.10[2].

Specifically, I note that the Defendant has made a career out of breaking into people's homes. While technically burglary is a crime against property, it is regarded as one of the more serious crimes because it is an intimate invasion,

striking a person where he or she is most vulnerable and least expecting to be disturbed in their privacy and integrity. Even when a person is not present when a criminal enters the home, a burglary victim often feels violated quite out of proportion to the value of any property taken or destroyed.

Mr. Ortiz's career as a burglar has spanned years, involving fifty arrests, nineteen convictions, and multiple parole violations. And as both defense counsel and the prosecutor point out, Defendant has pursued his life largely as a drug addict, to the point where he is now gravely ill. Extensive medical testimony and other evidence established that it is questionable whether, even if I sentence him merely as a second felony offender to the minimum sentence of 2 to 4 years, he would still be alive at the expiration of his sentence. Given his weakened state, any assertion that Defendant is a serious threat to the public as a burglar is really not credible. However, if released, he may well return to the life of a street addict, thereby endangering the public through the exchange or sharing of dirty needles. Moreover, I do find that he is certainly deserving of the enhanced punishment the statute permits, and thus I further find that lifetime supervision is totally appropriate.

Even the minimum sentence of 15 years to life will ensure Mr. Ortiz gets the medical treatment he clearly needs, in a setting that will ensure that he takes his medications on schedule, and without resort to the use and sharing of dirty needles. Should he actually live long enough to be released prior to the minimum sentence, there will remain an umbrella of supervision which will protect not only the public,

but Mr. Ortiz, as well.

Accordingly, for all of the foregoing reasons, I choose to exercise my discretion and sentence the Defendant as a persistent felony offender. I sentence the Defendant to a term of not less than fifteen years, nor more than life imprisonment.

This shall constitute the Decision and Order of the Court.

Date: April 4, 2006

ENTER

Richard Lee Price, J.S.C.
